# Aberrant leukocyte telomere length in Birdshot Uveitis

**DOI:** 10.1371/journal.pone.0176175

**Published:** 2017-05-01

**Authors:** Nadia Vazirpanah, Fleurieke H. Verhagen, Anna Rothova, Tom O. A. R. Missotten, Mirjam van Velthoven, Anneke I. Den Hollander, Carel B. Hoyng, Timothy.R. D. J. Radstake, Jasper C. A. Broen, Jonas J. W. Kuiper

**Affiliations:** 1 Laboratory of Translational Immunology, department of Immunology, University Medical Center Utrecht, Utrecht, The Netherlands; 2 Department of Ophthalmology, Erasmus University Medical Center, Rotterdam, The Netherlands; 3 The Rotterdam Eye Hospital, Rotterdam, The Netherlands; 4 Department of Ophthalmology, Radboud University Medical Center, Nijmegen, the Netherlands; 5 Department of Clinical Immunology and Rheumatology, University Medical Center Utrecht, Utrecht, The Netherlands; 6 Ophthalmo-Immunology, Department of Ophthalmology, University Medical Center Utrecht, Utrecht, The Netherlands; Tulane University Health Sciences Center, UNITED STATES

## Abstract

**Purpose:**

Birdshot Uveitis (BU) is an archetypical chronic inflammatory eye disease, with poor visual prognosis, that provides an excellent model for studying chronic inflammation. BU typically affects patients in the fifth decade of life. This suggests that it may represent an age-related chronic inflammatory disease, which has been linked to increased erosion of telomere length of leukocytes.

**Methods:**

To study this in detail, we exploited a sensitive standardized quantitative real-time polymerase chain reaction to determine the peripheral blood leukocyte telomere length (LTL) in 91 genotyped Dutch BU patients and 150 unaffected Dutch controls.

**Results:**

Although LTL erosion rates were very similar between BU patients and healthy controls, we observed that BU patients displayed longer LTL, with a median of log (LTL) = 4.87 (= 74131 base pair) compared to 4.31 (= 20417 base pair) in unaffected controls (*P*<0.0001). The cause underpinning the difference in LTL could not be explained by clinical parameters, immune cell-subtype distribution, nor genetic predisposition based upon the computed weighted genetic risk score of genotyped validated variants in *TERC*, *TERT*, *NAF1*, *OBFC1* and *RTEL1*.

**Conclusions:**

These findings suggest that BU is accompanied by significantly longer LTL.

## Introduction

Birdshot Uveitis (BU) is an archetypical and clinically well-defined inflammatory eye disease (uveitis) that damages retina and choroid tissues commonly leading to visual deterioration[1]. Although the cause of BU is not understood, it is characterized by ocular infiltrating and circulating inflammatory T lymphocytes[2–5] and exclusively affects major histocompatibility complex human leukocyte antigen (HLA)-A29-positive individuals[6,7]. BU typically manifests in the fifth decade of life[8,9]. This might imply that BU is an disease characterized by age-related failure of immune regulation and progressive chronic subclinical inflammation, eventually leading to uveitis in genetically susceptible individuals[10,11].

Telomeres are tandem repeat regions at the ends of eukaryotic chromosomes that shorten with increasing age as part of the normal ageing process[12]. The relative length of cellular telomeres is therefore considered to be an index for cellular senescence. Excessive erosion of telomeres in leukocytes was suggested to indicate persistent replicative stress. In line with this, leukocytes from patients with chronic inflammatory diseases often display an increased rate of telomere erosion, likely to be caused by persistent inflammation or genetic susceptibility affecting telomere biology genes[12,13].

In this study, we investigated the leukocyte telomere length in a unique cohort of genotyped BU patients and unaffected healthy controls.

## Patients and methods

This study was performed in compliance with the guidelines of the Declaration of Helsinki and has the approval of Institutional Review Boards and ethical committee of the University Medical Center Utrecht. After signed inform consent, blood was obtained from 91 unrelated Dutch BU cases at the Department of Ophthalmology at the University Medical Center Utrecht, the Eye Hospital Rotterdam and Radboud University Nijmegen Medical Center, the Netherlands. 150 unrelated Dutch healthy controls all from European ancestry were used as controls (Table 1). The diagnosis of BU was based on international guidelines[14]. Disease duration was typically between 5–10 years.

**Table 1:** Demographics of discovery and replication cohorts investigated in this study.

** **	**Birdshot Uveitis**	**Unaffected Control**	***P* value**
*N*	91	150	
*Female/Male (ratio)*	69/22 (3.0)	122/28 (4.4)	0.259^c^
*Age (years)*	60.5 (9.07)	52.9 (7.16)	<0.0001
*Leukocyte Telomere Length (base pares)*	74460.48 (126832.15)	20310.00 (44573.73)	<0.0001
* *	**% of patients**	**% of unaffected controls**	** **
*No systemic treatment*	27^a^	100	
*Systemic Corticosteroids*	25^a^	0	
*IMT*	31^a^	0	
*Systemic Corticosteroids + IMT*	17^a^	0	
*Active uveitis*	20^b^	0	

^a^ IMT = immunomodulatory treatment

^b^ n = 71

^c^ n = 50.

The values are represented in Median (Standard deviation). The significance of the association between patients and unaffected control was tested using Chi-square test (categorical values) and Mann-Whitney U test (continuous variables).

### Measurements of absolute telomere length in leukocytes

Whole blood EDTA samples were obtained from all participants. DNA extraction was similar for cases and controls and conducted by automated magnetic bead-based DNA isolation protocols (PerkinElmer). DNA concentration was quantified by Qbit Fluorometric Quantitation (Thermofischer Scientific). Leukocyte Telomere Length (LTL) was measured by Bio-rad cfx-96 real-time qualitative polymerase chain reaction (qPCR) detection system in duplicates in two separate experiments.

Briefly, the length of telomeres—long repetitive hexamer (TTAGGG) sequences–can be accurately determined by using a calibration curve based on linear serial dilution of a synthetic 84-mer (14 consecutive TTAGGG sequences) oligonucleotide (Geneworks, Adelaide, Australia) with a predetermined molecular weight per reaction (60x10^-12^ gr of telomere oligomer or 1.36x10^9^oligomers). The total number of base-pares in the highest standard can be calculated as ((1.36x10^9^ molecules of oligomer) x (84 oligomer length) = 1.18x10^8^ kilo base-pares).

The relative telomere length per sample is extrapolated from serial dilutions of the synthetic standard in each qPCR measurement. Similarly, a synthetic standard was also designed for the single copy house-keeping gene 36B4.

Absolute telomere base-pares per genome are quantified by subdividing the total number of telomere base-pares from 36B4 (which has only one copy per gene) following; Telomere length/house-keeping gene = telomere length per genome [15,16]. LTL for each individual in the study are outlined in S1 Table.

### Isolation of mononuclear immune cell subsets

Peripheral blood mononuclear cells (PBMCs) of 9 BU patients and 15 matched healthy individuals (S2 Table) were sorted to obtain immune cell-subtypes separately. PBMCs were isolated through Ficoll (Ficoll-Paque Plus, GE Healthcare). In order to sort out the CD3^+^/CD56^-^/CD4^+^ for T helper lymphocytes, CD3^+^/CD56^-^/CD8^+^ for Cytotoxic T lymphocytes, CD19^+^/CD20^+^ for B lymphocytes, CD14^+^ monocytes, CD3^-^/CD56^+^ NK cells and CD123 (IL3RA)^+^ / CD304 (BDCA4)^+^ pDCs, Fluorescence Activated Cell Sorting (FACS) (FACSAria III, BD Biosciences) was implemented.

### *Human TERT* gene expression measurements

Human telomerase (*hTERT*) gene expression levels of the sorted cell subsets were quantified by qPCR (Taqman Beadchip, applied Biosystems) according to the specific protocol indicated by the manufacturer by Quantstudio (Biosystems). Gene expression was normalized to Glucuronidase Beta (*GUSB*) and Glyceraldehyde-3-Phosphate Dehydrogenase (*GAPDH*).

### Statistical analysis

IBM SPSS Statistics v20 (SPSS, Chicago, IL) and Graphpad Prism v6 (GraphPad Software, San Diego, California) were used for statistical analyses as indicated in the results section. The telomere length data were natural log transformed to achieve a normal distribution. The Spearman’s Rank-Order correlation was implied to test the association between leukocyte count of untreated patients and LTL. The blood leukocyte count (*10^^9^/L) was assessed in the clinic simultaneous to patients visit. We computed a weighted genetic risk score (wGRS) in the 91 BU cases using genotype data from these cases obtained in a previous genome-wide association study[17] to test the cumulative effect of validated SNPs associated with telomere length[18] compared to an equivalent number of Dutch controls[17]. We calculated a weighted mean of genotype dosage across seven established risk alleles (Table 2) by multiplying the number of risk alleles at each locus (0, 1, 2) for the corresponding OR per allele and then summing the products[17]. We used a T-test to assess the differences between the mean absolute values of wGRS.

**Table 2:** Seven validated SNPs^17^ used to compute the genetic risk score (GRS) in BU and controls.

				*Allele Frequency*			
Gene	Chromosome	SNP	Risk allele	BU	Controls	Odds ratio	*P* value*
TERC	3	rs10936599	T	0.28	0.23	1.30	0.12
TERT	5	rs2736100	A	0.55	0.52	1.16	0.36
NAF1	4	rs7675998	A	0.22	0.21	1.12	0.58
OBFC1	10	rs9420907	A	0.82	0.84	0.81	0.30
ZNF208	19	rs8105767	A	0.74	0.73	1.10	0.57
RTEL1	20	rs755017	A	0.86	0.89	0.78	0.28
ACYP2	2	rs11125529	C	0.89	0.88	1.25	0.35

The significance of the association between patients and unaffected control was tested using Chi-square test. (*P*<0.05).

## Results

Table 1 provides demographic characteristics of the BU and control cohorts. After quality control, we analysed LTL data in 91 cases and 150 controls (S1 Table). Gender was equally distributed between BU and controls (Chi-Square Test, *P* = 0.259). The mean ages (range) of patients with BU and controls were 60.6 (30–84) and 52.9 (31–59) years, respectively. Although the age groups of the cases and controls were generally matched, the mean age of the BU cohort was significantly higher (*P*<0.0001).

**Fig 1:**
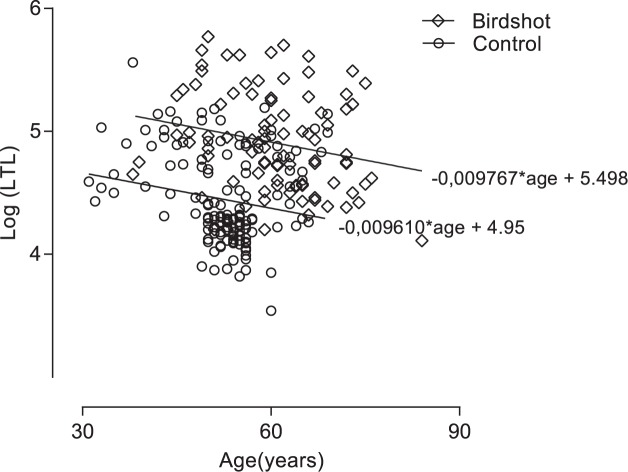
Dot plot showing natural log transformed leukocyte telomere length of Dutch BU patients (diamonds) and Dutch controls (empty circles).

**Fig 2:**
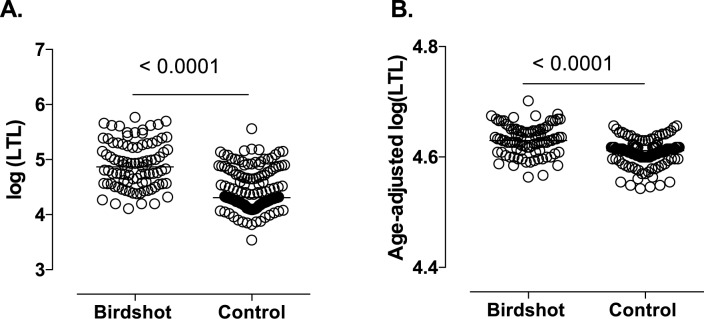
Median of log transformed leukocyte telomere length (kilo base-pairs) values represented on scatter dot plot showing **(A)** BU patients and healthy controls (*P* <0.0001, T-test) and **(B)** after adjustment for age (*P* <0.0001).

**Fig 3:**
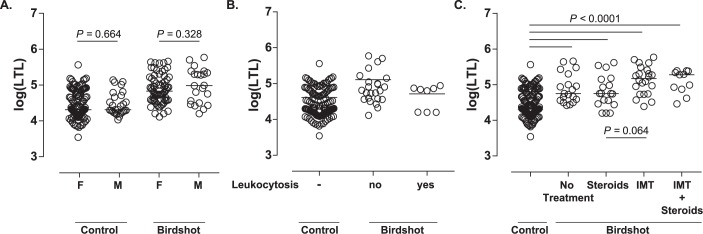
Median of natural log transformed leukocyte telomere length (kilo base-pairs) of BU patients and controls according to **(A)** gender (male (M) and female (F), *P* = 0.328) **(B)** presence of leucocytosis **(C)** and sytemic treatment (no treatment, (cortico)steroids, immune-modulatory treatment (IMT) and IMT + steroids).

The telomere length data were natural log transformed to achieve a normal distribution. As expected, a weak inverted linear correlation with age and LTL was demonstrated with LTL = -0.009767*age + 5.498; R^2^ = 0.045 (*P* = 0.04) for BU patients and LTL = -0.009610*age + 4.95; R^2^ = 0.038 (*P* = 0.02) for unaffected controls (Fig 1). Telomere length decreased with similar rates in BU patients and healthy participants (difference in slopes ~ *P* = 0.97) with an annual loss of [assuming (10^β1^–1)×100%] ~2.22% of telomere length in BU patients and 2.19% in controls. BU patients displayed longer LTL (in base pair [bp]) with a median of log (LTL) = 4.87 (= 74460 bp) compared to 4.31 (= 20310 bp) in the control group (*P*<0.0001, Fig 2). Age-adjusted telomere length computed by residuals derived from the linear model also supported significantly longer LTL (>3 times [bp]) in BU (median log (LTL) = 4.630 = 42658 bp) compared to controls (median log (LTL) = 4.216 = 13366 bp) (*P*<0.0001, Fig 3). In line with previous observations[19–21], age explained very little of the total variability of LTL (adjusted R^2^<0.04 in cases and controls, Fig 1). To avoid over-fitting due to large residuals, we conservatively did not correct for age in subsequent analyses.

The LTL was similar between men and women in both BU (median log (LTL) = 4.985 vs. 4.830, *P* = 0.328) and controls (4.310 vs. 4.310, *P* = 0.664, Fig 3A). We also did not observe a difference in LTL between patients with (n = 34) and without leucocytosis (n = 57) at the time of sampling (Fig 3B). To investigate if treatment affected LTL, we subdivided the patients according to available treatment data of 71 cases (Fig 3C). All of the treatment groups, including patients who did not have a history of systemic treatment displayed significantly longer LTL (Fig 3B). The mean LTL of patients treated with immune-modulatory treatment (IMT) was slightly higher compared to other treatment regimes, but did not reach statistical significance (*P>*0.06, Fig 3C).

We had the opportunity to study telomere length and expression of telomere biology genes in sorted immune cell subsets of 9 BU and 15 healthy individuals. LTL differences between CD4^+^ and CD8^+^ T lymphocytes, CD19^+^ B lymphocytes, CD14^+^ monocytes, CD3-/CD56+ natural killer (NK) cells and CD123+/CD304+ plasmacytoid dendritic cells (pDC) of BU patients and healthy individuals did not reveal statistically significant differences in telomere length (S2 Table). Also, in contrast to house-keeping genes (*GUSB* and *GAPDH*), *hTERT* gene expression was not detected in these cell subtypes (S1 Fig). Six of these BU patients were naïve to systemic treatment and revealed slightly higher leukocyte count and a positive correlation (r = 0.551, *P*>0.05) between leukocyte count and LTL. However, this observation is underpowered due to low number of untreated patients in which leukocyte count is quantified.

**Fig 4:**
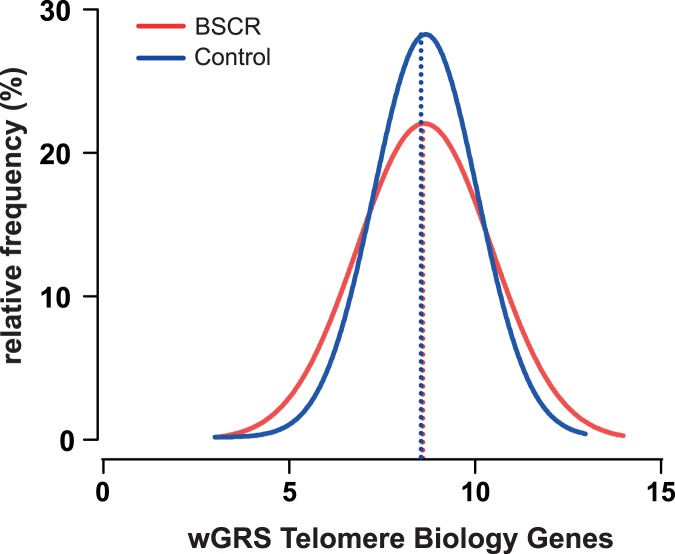
Distribution of the relative frequency of the weighted genetic risk score (wGRS) of validated variants in telomere biology genes (Table 2.) of BU patients (red line) (mean wGRS [range] = 8.598[3.650–13.34]) and healthy control participants (blue line) (mean wGRS [range] = 8.552[3.740–12.29]). Difference between BU and healthy control participants was tested via t- test (*P* = 0.859).

Seven validated genetic variants that are known to be involved in telomere biology (*TERC*, *TERT*, *NAF1*, *OBFC1* and *RTEL1*) have been shown to significantly affect telomere-length in age-related diseases[18]. Because BU displayed longer LTL, we investigated potential genetic predisposition of these known risk loci in BU that could explain the difference in LTL. Therefore, we computed a weighted genetic risk score (wGRS) based on the observed number of risk alleles per case or control and adjusted their effect sizes based on the previous GWAS of BU[17]. The allele frequencies in cases and controls were very similar to the previous meta-analyses by Codd et al, indicating that the allele distribution reflects larger populations and can be used for good estimate of the GRS[18] (Table 2). The mean wGRS was highly similar between BU and controls (mean wGRS[range] = 8.598[3.650–13.34] for BU and 8.552[3.740–12.29] for controls, t-test: *P* = 0.859, (Fig 4). Also, the risk alleles did not show consistency in effect direction of BU patients (in other words, positive and negative odds ratios, Table 2). These analyses suggest that there is no genetic predisposition for telomere length in BU patients. Based upon these findings we conclude that the increased LTL can be attributed to disease specific mechanisms.

## Discussion

BU is a severe chronic inflammation of the posterior eye segment that damages ocular tissues resulting in visual impairment. BU typically affects middle-aged and elderly individuals of European descent[1]. Hallmark of disease is the appearance of the distinctive ‘birdshot’ pattern of multiple white spots on the fundus, from which it obtained its name[22].

In the current study we show that leukocytes from BU patients have longer telomere length than population-based controls. Despite extensive efforts, the mechanism behind this observation is currently enigmatic. The telomere measurement method we used has been well described in the literature and is known for its high reproducibility[15]. In contrast to the common use of reference samples derived from a patient, control, or cell line, we optimized reproducibility by exploiting synthetic telomere repeats and 36B4 gene copies as standards. A great advantage of using stable synthetic standards is the possibility for absolute quantification of telomere lengths[16]. Since cases and control samples have been measured twice in random order by the same qPCR machine, settings and reagents, we are confident that the difference in LTL is unrelated to methodology.

Telomere length measurements in several immune cell-subtypes did not reveal overall changes in telomere length of leukocytes and may be the result of a yet un sampled immune cell-subset or would need larger sample size to investigate this at the cell subset level[23,24]. BU is a rare disease and biosamples are scarce making it hard to obtain a large patient cohort[25], thus, we were only able to include a limited number of patients for cell sorting. Recent studies demonstrated changes telomere length are better monitored in longitudinal studies following participants over a longer time course[26]. Accordingly, telomere length quantification in following studies overtime might unravel clues on telomere biology of BU patients.

Previous studies have mapped putative loci known to be involved in telomere biology and demonstrated association of 7 lead variants in 5 telomere regulatory genes (Table 2) with LTL in age-related diseases[18]. This causal role of genetic predisposition in LTL led us to investigate if the much longer telomeres were caused by genetic predisposition in the BU patients. When we applied our previously published whole genome data to the trait of having longer telomeres in the BU population, we did not observe cumulative effect of variants linked to telomere length in these patients. Thus, it is reasonable to assume that there is no genetic predisposition for telomere length in BU patients. Although it is possible that the sample size and power were too low to detect such an association, the highly similar genetic risk score in cases and controls and the lack of consistent direction of effect advocate for very low (if any) contribution of genetic variants to the large difference of LTL between BU and healthy controls. This observation, in addition to the fact that both patients and controls share the same ethnic background (cases and controls are all Dutch Caucasians from European ancestry) makes the longer telomeres unlikely the result of population stratification. Based upon these findings we conclude that the increased LTL can be attributed to BU-specific mechanisms.

When we analysed our data from a population/demographic perspective, it appears that the population of BU patients investigated in this study is slightly older compared to the healthy individuals. However, when we applied age-correction based on the incremental slopes of telomere shortening with age, the changes in LTL remained significant. Since we consider methodological, genetic and demographic factors to be minimally involved, we assume disease mechanisms are driving the aberrant LTL.

Whether the longer LTL is a consequence of BU or contributes to disease onset remains to be elucidated. The present study was cross-sectional and involved measurement of telomere length at only one time point, and no data on telomere erosion rates are available. Furthermore, telomere length can be affected by various metabolic and biological factors [27]. The general view of chronic inflammation is that it is accompanied by shortening of leukocyte telomere length due to replicative stress[28,29]. Yet, increased LTL has been reported in several chronic inflammatory diseases[30] such as rheumatoid arthritis (RA) and ankylosing spondylitis. Curiously, like BU, the latter also strongly linked to HLA class I (HLA-B27)[31,32]. In fact, comparable studies in *systemic lupus erythmatodes* and RA demonstrated that longer telomere length was particularly observed in older patients–similar to the representative age of onset in BU[33,34].

In addition to its clinically well-described manifestations, BU is renowned outside the field of ophthalmology for its unusually strong link with the HLA-A29. Essentially all patients are HLA-A29 positive, which represents the strongest associations between an HLA class I allele and human disease[35,36]. Nevertheless, the role of HLA-A29 has not yet been elucidated and consequently the pathophysiology of BU is not well understood[1]. However, in recent years an emerging line of evidence is beginning to yield clues on the disease biology of BU. The recent genome-wide analysis of BU confirmed the extreme association with HLA-A29:02 and revealed a strong link with the *endoplasmic reticulum aminopeptidase 2* (ERAP2) gene, indicating that peptide processing in the endoplasmic reticulum and presentation to T cells are the key mechanism of the disease[3]. Indeed, T cells are the dominant infiltrating cells found in the eyes of patients[2]. The ocular microenvironment and blood of patients display elevated levels of IL-17-related cytokines and an increased frequency of circulating T helper 17 and T cytotoxic 17 cells. These T cell subsets are considered as important pathogenic drivers of various chronic inflammatory disorders[5,37–39].

Another explanation might be that activated naïve T cells are able to up-regulate telomerase expression, although this ability remains controversial[26,40–42]. In highly proliferative cells such as stem cells, germ cells and many neoplastic cells, telomerase is decisive for telomere maintenance. In T cells, despite being normal somatic cells, telomerase expression during proliferation and an elevated telomerase activity was demonstrated in immature naïve cells as compared to mature thymocytes, and was almost undetectable in mature resting naïve T cells[43,44]. Although we did not observe increased telomerase expression in T cells in BU, we did not make any distinction between the naïve and the memory T cell compartment within the examined immune-cell panel. Interestingly, CD8+ antigen-specific T cells display more robust inflammatory responses in individuals with longer telomere length[45] and CD4+ antigen-specific T cells have relatively longer telomere length compared to naïve cells[46]. Since BU patients show retinal autoimmunity, reflected by enhanced T cell proliferation towards retinal antigens[36,47,48], the increased LTL could reflect enrichment for ocular-specific T cells that drive chronic inflammation in BU. If longer telomere length in T cells confers risk in HLA-A29 positive individuals for developing BU, further investigation is needed that includes HLA-A29 positive controls. Alternatively, LTL may also be the result of other immune cell subsets. Interestingly, plasma cell differentiation from B cells–the antibody-producing and T cell-activating B cell population–was reported to be accompanied by a significant elongation of telomeres of these cells[49]. Inline with this, our results indicate longer telomeres in B cells of BU patients (S2 Table), despite the limited sample size and consequential lack of power, this is an important observation since anti-retinal antibodies are emerging as important contributors in the pathophysiology of uveitis.

The complex dynamics of telomere biology in chronic inflammatory and autoimmune diseases advocates for detailed dissection of multiple epigenetic mechanisms in distinct cell subsets.

In conclusion, BU patients show longer telomeres compared to healthy controls which implies sophisticated telomere biology in chronic inflammation that warrants further research into the leukocyte populations involved in BU.

## Supporting information

S1 Fig The *hTERT* gene expression levels in cell subsets from (corrected to the house-keeping genes–see methods) BU patients (diamonds) and Dutch controls (HC, empty circles). (TIFF)

S1 Table The leukocyte telomere length (bp) measured by qPCR (see methods) for each Birdshot Uveitis patients and unaffected Dutch control. (DOCX)

S2 Table Telomere length in immune cell subsets of BU and controls. Telomere length (base pairs) and age (years) are represented in Median (Standard deviation).
(DOCX)
