# Supplementary material for: Aberrant leukocyte telomere length in Birdshot Uveitis
Source: PLoS One. 2017 May 1;12(5):e0176175. doi: 10.1371/journal.pone.0176175 (PMC5411068; doi:10.1371/journal.pone.0176175)
Supplement: S1 Table — (DOCX) [file pone.0176175.s002.docx]

**S1 Table. The leukocyte telomere length (bp) measured by qPCR (see methods) for each Birdshot Uveitis patients and Unaffected Dutch Control.**

| *Birdshot Uveitis case* | *Leukocyte Telomere length* | *Unaffected Control* | *Leukocyte Telomere length* |
| --- | --- | --- | --- |
| 1 | 68150 | 1 | 18802 |
| 2 | 62750 | 2 | 102754 |
| 3 | 26833 | 3 | 20863 |
| 4 | 12996 | 4 | 15265 |
| 5 | 21074 | 5 | 75472 |
| 6 | 165792 | 6 | 31473 |
| 7 | 99360 | 7 | 12672 |
| 8 | 95941 | 8 | 26642 |
| 9 | 36048 | 9 | 18308 |
| 10 | 24256 | 10 | 3498 |
| 11 | 81450 | 11 | 72849 |
| 12 | 52658 | 12 | 23665 |
| 13 | 80661 | 13 | 20764 |
| 14 | 54625 | 14 | 360163 |
| 15 | 18745 | 15 | 7068 |
| 16 | 24267 | 16 | 18303 |
| 17 | 37689 | 17 | 136571 |
| 18 | 35796 | 18 | 104259 |
| 19 | 65231 | 19 | 19366 |
| 20 | 62901 | 20 | 16557 |
| 21 | 39719 | 21 | 138456 |
| 22 | 46176 | 22 | 17565 |
| 23 | 111346 | 23 | 19841 |
| 24 | 245108 | 24 | 18225 |
| 25 | 24827 | 25 | 67752 |
| 26 | 55111 | 26 | 19201 |
| 27 | 203372 | 27 | 13573 |
| 28 | 90151 | 28 | 68394 |
| 29 | 245162 | 29 | 16644 |
| 30 | 85360 | 30 | 19461 |
| 31 | 191733 | 31 | 16837 |
| 32 | 28844 | 32 | 17567 |
| 33 | 218509 | 33 | 12448 |
| 34 | 42101 | 34 | 76151 |
| 35 | 307473 | 35 | 145383 |
| 36 | 201504 | 36 | 17623 |
| 37 | 123046 | 37 | 13173 |
| 38 | 93696 | 38 | 61720 |
| 39 | 166919 | 39 | 35859 |
| 40 | 418267 | 40 | 11792 |
| 41 | 37263 | 41 | 141524 |
| 42 | 74460 | 42 | 30401 |
| 43 | 405548 | 43 | 19806 |
| 44 | 590091 | 44 | 81748 |
| 45 | 32485 | 45 | 7614 |
| 46 | 97594 | 46 | 19081 |
| 47 | 193013 | 47 | 119112 |
| 48 | 241076 | 48 | 12530 |
| 49 | 256527 | 49 | 80039 |
| 50 | 186998 | 50 | 26198 |
| 51 | 112866 | 51 | 28586 |
| 52 | 54164 | 52 | 24925 |
| 53 | 133804 | 53 | 102047 |
| 54 | 454188 | 54 | 12792 |
| 55 | 440384 | 55 | 12763 |
| 56 | 302814 | 56 | 15562 |
| 57 | 56820 | 57 | 31106 |
| 58 | 56010 | 58 | 49292 |
| 59 | 45066 | 59 | 9112 |
| 60 | 27502 | 60 | 15208 |
| 61 | 39103 | 61 | 33254 |
| 62 | 100695 | 62 | 7477 |
| 63 | 200727 | 63 | 35845 |
| 64 | 347877 | 64 | 10643 |
| 65 | 176430 | 65 | 19462 |
| 66 | 31965 | 66 | 46217 |
| 67 | 417357 | 67 | 34324 |
| 68 | 55871 | 68 | 13277 |
| 69 | 91113 | 69 | 12740 |
| 70 | 15945 | 70 | 23510 |
| 71 | 15784 | 71 | 102417 |
| 72 | 15826 | 72 | 14052 |
| 73 | 72890 | 73 | 78415 |
| 74 | 87272 | 74 | 38835 |
| 75 | 502429 | 75 | 15885 |
| 76 | 53631 | 76 | 47924 |
| 77 | 269373 | 77 | 76408 |
| 78 | 141246 | 78 | 52335 |
| 79 | 54712 | 79 | 90336 |
| 80 | 31706 | 80 | 16050 |
| 81 | 36424 | 81 | 11596 |
| 82 | 150583 | 82 | 20265 |
| 83 | 306056 | 83 | 25523 |
| 84 | 57071 | 84 | 16312 |
| 85 | 27489 | 85 | 14323 |
| 86 | 85003 | 86 | 16441 |
| 87 | 96454 | 87 | 17989 |
| 88 | 37435 | 88 | 79389 |
| 89 | 35946 | 89 | 156629 |
| 90 | 28724 | 90 | 64745 |
| 91 | 26425 | 91 | 21458 |
|  |  | 92 | 90655 |
|  |  | 93 | 20866 |
|  |  | 94 | 12211 |
|  |  | 95 | 33145 |
|  |  | 96 | 83086 |
|  |  | 97 | 152389 |
|  |  | 98 | 8030 |
|  |  | 99 | 98452 |
|  |  | 100 | 44300 |
|  |  | 101 | 77392 |
|  |  | 102 | 19793 |
|  |  | 103 | 19406 |
|  |  | 104 | 89579 |
|  |  | 105 | 107036 |
|  |  | 106 | 44280 |
|  |  | 107 | 17521 |
|  |  | 108 | 15150 |
|  |  | 109 | 20538 |
|  |  | 110 | 46571 |
|  |  | 111 | 39386 |
|  |  | 112 | 11133 |
|  |  | 113 | 12883 |
|  |  | 114 | 14733 |
|  |  | 115 | 16871 |
|  |  | 116 | 12192 |
|  |  | 117 | 15095 |
|  |  | 118 | 10489 |
|  |  | 119 | 84001 |
|  |  | 120 | 13141 |
|  |  | 121 | 7431 |
|  |  | 122 | 15758 |
|  |  | 123 | 123831 |
|  |  | 124 | 12075 |
|  |  | 125 | 52636 |
|  |  | 126 | 124083 |
|  |  | 127 | 13968 |
|  |  | 128 | 9871 |
|  |  | 129 | 25759 |
|  |  | 130 | 62281 |
|  |  | 131 | 53337 |
|  |  | 132 | 20355 |
|  |  | 133 | 19451 |
|  |  | 134 | 50953 |
|  |  | 135 | 24990 |
|  |  | 136 | 45492 |
|  |  | 137 | 6543 |
|  |  | 138 | 8858 |
|  |  | 139 | 29240 |
|  |  | 140 | 18972 |
|  |  | 141 | 19171 |
|  |  | 142 | 16898 |
|  |  | 143 | 12633 |
|  |  | 144 | 20505 |
|  |  | 145 | 16244 |
|  |  | 146 | 20940 |
|  |  | 147 | 16115 |
|  |  | 148 | 58688 |
|  |  | 149 | 20243 |
|  |  | 150 | 16966 |
