# Supplementary material for: Aberrant leukocyte telomere length in Birdshot Uveitis
Source: PLoS One. 2017 May 1;12(5):e0176175. doi: 10.1371/journal.pone.0176175 (PMC5411068; doi:10.1371/journal.pone.0176175)
Supplement: S2 Table — Telomere length (base pairs) and age (years) are represented in Median (Standard deviation). (DOCX) [file pone.0176175.s003.docx]

**S2 Table**. **Telomere length in immune cell subsets of BU and controls.**

|  | **Birdshot Uveitis** | **Unaffected Control** | ***P* value** |
| --- | --- | --- | --- |
| *N* | 9 | 15 |  |
| *Female/Male (ratio)* | 3/6 (0.5) | 4/11(0.4) | 0.259 |
| *Age (years)* | 54 (11) | 50 (9) | <0.0001 |
| *Telomere length CD3+/CD4+* | 4902.78 (2258) | 6439.00 (4501) | 0.5421 |
| *Telomere length CD3+/CD8+* | 3827.00 (11764) | 5046.00 (4841) | 0.0821 |
| *Telomere length CD19+/CD20-* | 8910.78 (3111) | 6771.13 (5822) | 0.8374 |
| *Telomere length CD14+/CD16-* | 3797.75 (2478) | 6109.25 (4375) | 0.1448 |
| *Telomere length CD3-/CD56+* | 4169.00 (1232) | 4748.86 (1934) | 0.2047 |
| *Telomere length CD123+ / CD304+* | 6853.33 (2405) | 3250.14 (4469) | 0.1274 |

Telomere length (base pairs) and age (years) are represented in Median (Standard deviation).
